# Ras signaling regulates osteoprogenitor cell proliferation and bone formation

**DOI:** 10.1038/cddis.2016.314

**Published:** 2016-10-13

**Authors:** Garyfallia Papaioannou, Fatemeh Mirzamohammadi, Tatsuya Kobayashi

**Affiliations:** 1 Massachusetts General Hospital and Harvard Medical School, Boston, MA, USA

## Abstract

During endochondral bone development, osteoblasts are continuously differentiated from locally residing progenitor cells. However, the regulation of such endogenous osteoprogenitor cells is still poorly understood mainly due to the difficulty in identifying such cells *in vivo*. In this paper, we genetically labeled different cell populations of the osteoblast linage using stage-specific, tamoxifen-inducible Cre transgenic mice to investigate their responses to a proliferative stimulus. We have found that overactivation of Kras signaling in type II collagen-positive, immature osteoprogenitor cells, but not in mature osteoblasts, substantially increases the number of their descendant stromal cells and mature osteoblasts, and subsequently increases bone mass. This effect was mediated by both, the extracellular signal-regulated kinase (ERK) and phosphoinositide 3 kinase (PI3K), pathways. Thus we demonstrate that Ras signaling stimulates proliferation of immature osteoprogenitor cells to increase the number of their osteoblastic descendants in a cell-autonomous fashion.

Bones are formed and maintained by multiple types of bone cells.^1^ Mineralized bone matrix is produced by osteoblasts. Osteoblasts produce a large amount of bone matrix proteins including type I collagen and osteocalcin. Osteoblasts then die or further differentiate into osteocytes that are embedded in the bone matrix.^2, 3^ Bone is constantly remodeled throughout life. During this process, osteoblasts are continuously differentiated from locally residing mesenchymal progenitor cells. Thus, proliferation and differentiation of the osteoblast progenitors need to be well coordinated to maintain adequate bone mass and function.

However, characteristics, kinetics and regulation of such endogenous osteoprogenitor cells are still poorly understood, primarily due to the lack of proper tools for *in vivo* analysis of such cells.

Recently, it has been shown that a subset of early osteoprogenitor cells show a promoter activity of the type II collagen gene (Col2a1); a lineage tracing study using Col2-creER and Cre reporter transgenic mice in which tamoxifen injection genetically labels Col2-positive cells shows that Col2-positive cells include self-renewing early osteoprogenitor cells.^4^ Upon lineage commitment, osteoprogenitor cells start expressing Sp7 (Osterix; Osx).^3, 4, 5^ In contrast, mature osteoblasts and their descendants can be exclusively labeled by the use of short promoter fragments of the type I collagen gene (*Col1a1*).^3, 6^

In this study, using stage-specific, tamoxifen-inducible Cre transgenic lines, we demonstrate that Ras signaling regulates proliferation of Col2-positive osteoprogenitor cells and controls bone mass, whereas Ras overactivation shows little effect in mature osteoblasts. Thus, the difference of responsiveness to Ras signaling discriminates differentiation stages of cells of the osteoblast lineage.

## Results

### Col2-positive cells are self-renewing osteoprogenitors

**Figure 1:**
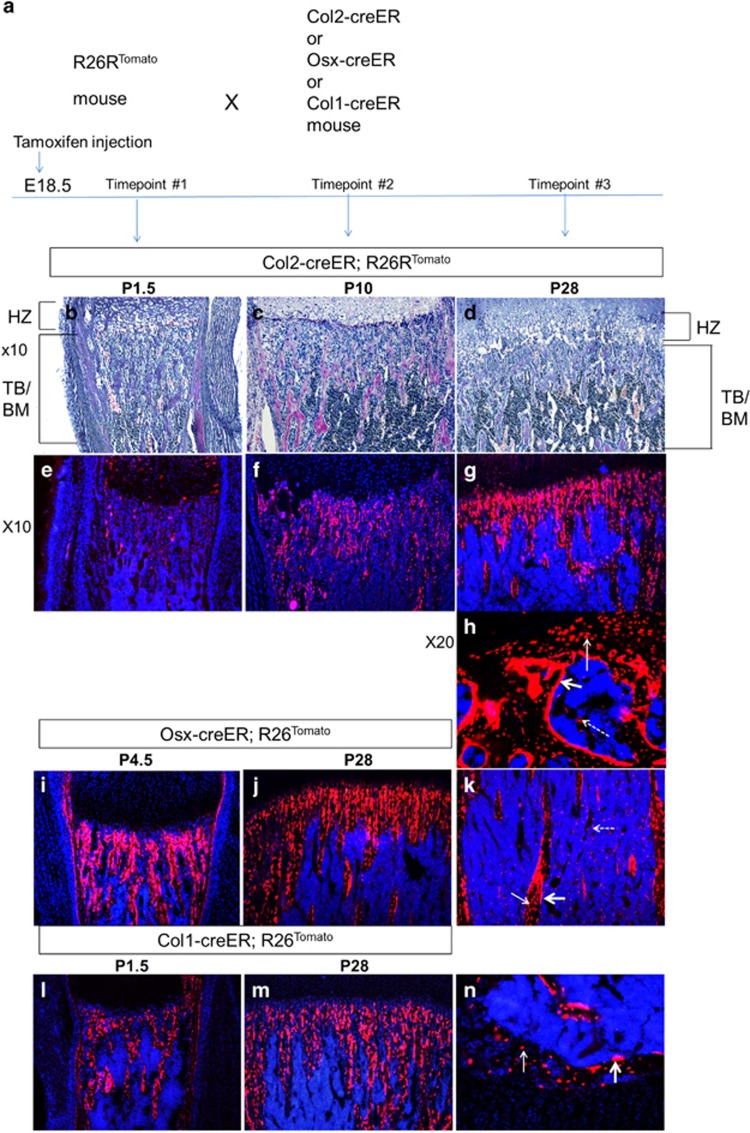
The fate and localization of bone-forming cells: Col2-, Osx- and Col1-positive cells during bone development (see also Supplementary Figure S1). (**a**) Experimental design. R26R-tdTomato reporter mice were crossed with transgenic mice expressing creER under the control of Col1, Osx and Col2 promoters. A single dose of tamoxifen (0.1 mg/g) was intraperitoneally (i.p.) injected into pregnant mothers at E18.5. (**b**-**d**) Hematoxylin/eosin-stained paraffin sections of the proximal metaphysis of the tibia at indicated ages (P=postnatal day, TB=trabecular bone, BM=bone marrow, HZ=hypertrophic chondrocyte zone). (**e**-**g**) Fluorescent images of the tibia of B-D (× 10). Blue:Dapi; Red:Tomato red fluorescent protein. (**h**) A cryosection (× 20 magnification) of the secondary ossification center in the Col2-creER;R26R^tomato^ mouse tibia at P28. Tomato-labeled cells are observed on the bone surface (osteoblasts; thick arrow), inside of the bone matrix (osteocytes; thin arrow), and in the bone marrow (dotted arrow). (**i**-**j**) Cryosections (× 10 magnification) of the Osx-creER;R26R^tomato^ mouse tibiae at indicated ages. Blue, DAPI; red, Tomato red fluorescent protein. (**k**) Cryosection (× 10 magnification) of the Osx-creER;R26R^tomato^ mouse tibia bone marrow at P28. Blue: DAPI; red: Tomato red fluorescent protein; thick arrow: osteoblasts; thin arrow: osteocytes; dotted arrow: stromal cells. (**l**-**m**) Cryosections (× 10 magnification) of the Col1-creER;R26R^tomato^ mouse tibia pulsed with tamoxifen at age E18.5. Tomato (red fluorescent protein) labeled the Col1-positive cells at age E18.5 and cell fate was chased at ages P1.5 and P28. (**n**) Cryosection of Col1-creER;R26R^tomato^ mouse tibia showing the secondary ossification center (× 20 magnification) at P28. Blue, DAPI; red, Tomato red fluorescent protein. thin arrow: osteocytes; thick arrow: osteoblasts. No tomato-positive cells were found in the bone marrow. *n*=5 mice per group

To determine the fate of osteoblast-lineage cells in different differentiation stages, we genetically labeled cells using stage-specific, tamoxifen-inducible Cre transgenic mice and R26R-tdTomato Cre reporter mice. We crossed R26R-tdTomato reporter mice^7^ with Cre transgenic mice expressing tamoxifen-inducible Cre recombinase driven by a type II collagen (*Col2a1*) promoter (Col2-creER),^8^ an osterix (*Sp7; Osx*) promoter (Osx-creER),^9^ or a 3.2 kb-long type I collagen promoter (Col1-creER)^9^ (Figure 1a). We injected a single dose of tamoxifen into pregnant mothers at E18.5 to label Cre-expressing cells. Tamoxifen induces Cre recombination and subsequently tomato expression, and thus Cre-expressing cells as of tamoxifen injection and their descendants will express tomato red fluorescent protein.

As recently shown in postnatal mice,^4^ a small number of Col2-creER-expressing (Col2-positive) cells were labeled in the bone marrow 2 days after tamoxifen injection (Figures 1b and e, Supplementary Figures S1A, S1D). Over time, these cells produced a significant number of descendants that differentiated into bone stromal cells, osteoblasts and osteocytes (Figures 1c, Supplementary Figures S1B, S1C, S1E, S1F).

As expected, Osx-creER-expressing (Osx-positive) and Col1-creER-expressing (Col1-positive) cells were abundantly present on bone surface at early time points after tamoxifen injection. In these models, numbers and localization patterns of RFP-labeled cells appeared relatively constant during the 1-month chase period (Figures 1i and n, Supplementary Figures S1G-S1N). Col1-creER-expressing cells and their descendants are mainly osteoblasts and osteocytes (Figure 1n, Supplementary Figure S1N), whereas Osx-creER labels stromal cells, osteoblasts and osteocytes (Figure 1k, Supplementary Figure S1J). Tamoxifen injection into Osx-creER at E18.5 does not label cells in the secondary ossification center (Supplementary Figure S1H). Because the secondary ossification center is not yet formed at E18.5, this finding suggests that Osx-positive cells and their descendants in the primary ossification center unlikely migrate into the secondary ossification center to serve as a source of osteoblasts.

### Expression of an oncogenic Kras in Col2-positive cells increases the number of their descendant cells and trabecular bone mass

Mechanisms that regulate proliferation, differentiation and maintenance of endogenous osteoprogenitor cells in bone are still largely unknown. The recent finding that a self-renewing endogenous osteoprogenitor population can be genetically labeled using Col2-creER and R26R-tdTomato transgenic mice provided an opportunity to investigate the regulation of osteoprogenitor cells *in vivo*.

One of major regulators of cell proliferation is the Ras signaling pathway. The small GTPase, Ras, mediates the extracellular proliferative stimuli to downstream intracellular signaling molecules, including the extracellular signal-regulated kinase (ERK) and phosphoinositide 3 kinase (PI3K) pathways, to stimulate cell cycle progression. Thus, we hypothesized that Ras-mediated signaling pathways might regulate osteoprogenitor cell proliferation.

In order to study the effect of overactivation of Ras signaling in osteoprogenitor cells *in vivo*, we expressed an active form of Kras (Kras^G12D^)^10^ in Col2-positive cells that were simultaneously labeled with Tomato red fluorescent protein using Col2-creER transgenic mice. Expression of Kras^G12D^ and Tomato was induced at E18.5 by a single dose of tamoxifen injection into pregnant mothers.

**Figure 2:**
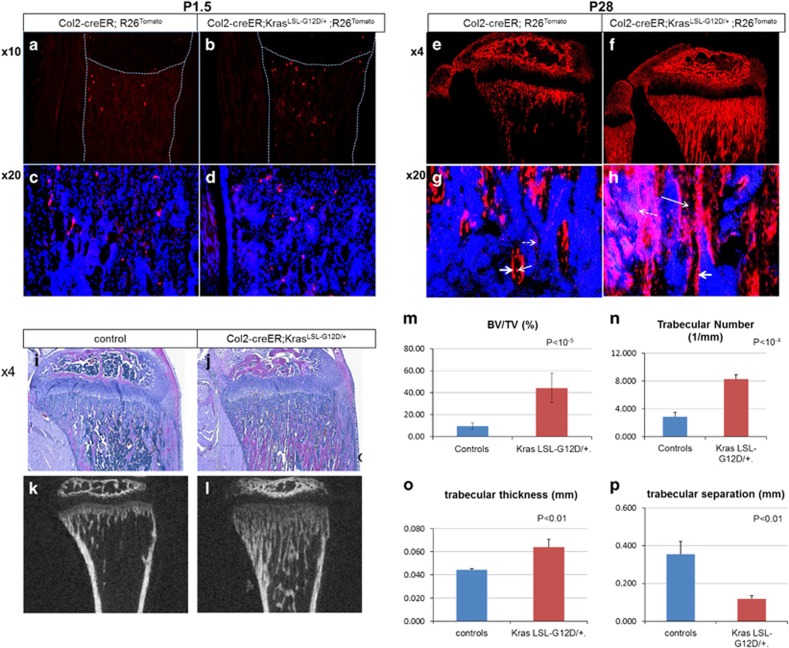
Expression of Kras^G12D^ in Col2-positive cells at E18.5 increases the number of their descendants and the trabecular bone mass (see also Supplementary figure S2). (**a**-**d**) Tomato-positive cells in the tibial metaphysis of Col2-creER;R26R^tomato^ mice with wild-type Kras (**a**, **c**) and Kras^G12D^ (**b**,**d**) at P1.5. Mice were treated with tamoxifen at E18.5. A,B: × 4 magnification. **c**,**d**: × 20 magnification. Blue, DAPI; red, Tomato red fluorescent protein. Dotted lines indicate the borders of tibia. The middle dotted line indicates the border between growth plate and primary spongiosa (trabecular bone). Magnifications of primary spongiosa of **a** and **b** are shown in **c** and **d** respectively (*n*=3). (**e**-**h**) Tomato-positive cells in the tibial metaphysis of Col2-creER;R26R^tomato^ mice with wild-type Kras (**e**,**g**) and Kras^G12D^ (**f**,**h**) at P28. The mice were treated with tamoxifen at E18.5. E,F: × 4 magnification. **g**,**h**: × 20 magnification. Blue, DAPI; red, Tomato red fluorescent protein; arrow: osteocytes; double arrow: osteoblasts; dotted arrow: stromal cells (*n*=4). (**i**-**j**) Hematoxylin/eosin-stained paraffin sections of tibias at P28 from Col2-creER mice with wild-type Kras (**i**) and Kras^G12D^ (**j**) after Kras activation at E18.5 (× 4 magnification). Mice harboring Kras^G12D^ show increased trabecular bone with a minimal effect in the growth plate structure (*n*=8). (**k**-**l**) MicroCT pictures of tibias at P28 from Col2-creER mice with wild-type Kras (**k**) and Kras^G12D^ (**l**) after Kras activation at E18.5. (**m**-**p**) Graphs showing comparisons of bone volume fraction (**m**), trabecular number (**n**), trabecular thickness (**o**) and trabecular separation (**p**) measured by microCT in mice with wild-type Kras or Kras^G12D^ at P28 (*n*=3 and *n*=4 for mutant and control groups, respectively)

Two days after tamoxifen injection, only small numbers of Tomato-expressing cells were found in the metaphyseal region of both Kras^G12D^-positive and negative mice (Figures 2a and d). However, 1 month later Tomato-positive cells were significantly more abundant in Kras^G12D^-positive mice than in Kras^G12D^-negative mice (Figures 2e and f). The increase in Tomato-positive cells in Kras^G12D^-positive mice involved osteocytes, osteoblasts as well as stromal cells (Figures 2g and h). Hematoxylin-eosin (H/E) staining of tibia sections showed a striking increase in trabecular bone mass in mice with Kras activation in Col2-positive cells and their descendants (Figures 2i and j). Littermates lacking Cre or Kras were used as controls, and they received the same amount of tamoxifen as the mutant mice. Kras^G12D^ mice without Cre recombination had the same bone phenotype as wild-type mice. In the absence of tamoxifen there were no overt bone phenotypes in mutant mice (Supplementary Figures S2A-S2B).

A similar phenotype was observed in the humerus as in the tibia upon Kras activation in Col2-postive cells (Supplementary Figures S2C-S2D). Microcomputed tomography (*μ*CT) analysis showed that there was a significant increase in the bone volume fraction, trabecular number and trabecular thickness and a decrease in trabecular separation (Figures 2k and p). The cortical bone parameters were unchanged. There were no overt changes in tartrate-resistant acid phosphatase (TRAP) staining (Supplementary Figures S2I-S2J), suggesting that bone-resorbing osteoclasts were not affected.

**Figure 3:**
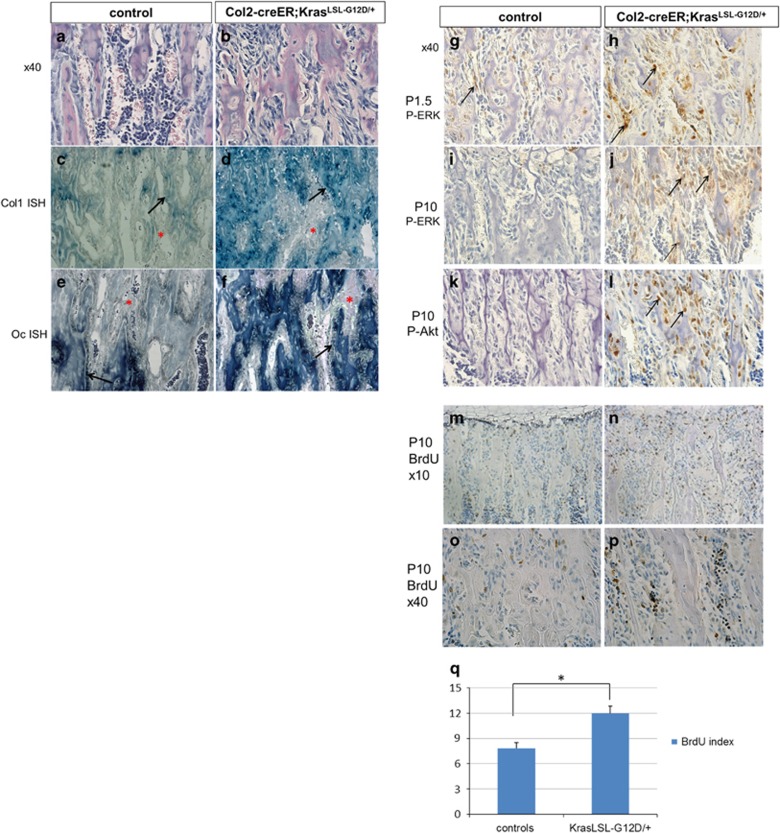
Activation of Kras oncogene in Col2 cells at perinatal age increases stromal cell numbers (see also Supplementary figure S3). (**a**-**b**) Hematoxylin/eosin-stained paraffin sections showing the stromal cells between the trabeculae in P28 mice (secondary spongiosa) control (**a**) and Kras^G12D^ (**b**) after Kras activation at E18.5. × 40 magnification. (**c**-**f**) *In situ* hybridization for collagen 1 (Col1 ISH) (**c**,**d**) and osteocalcin (Oc ISH) (**e**,**f**) in the secondary spongiosa of P28-old mice control (**c**,**e**) or Kras^G12D^ (**d**,**f**) after tamoxifen injection at E18.5. Stromal cells are not stained for these markers. Black arrows, osteoblasts; red asterisks, stromal cells. (**g**-**j**) Immunohistochemistry for p-ERK at P1.5 (**g**,**h**) and P10 (**i**,**j**) in the tibia secondary spongiosa of control (**g**, **i**) and Kras^G12D^ (**h**, **j**) after tamoxifen injection at E18.5. Arrows show representative cells stained with anti- p-ERK. (**k**-**l**) Immunohistochemistry for p-Akt at P10 in the tibia secondary spongiosa of control (**k**) or Kras^G12D^ (**l**) after tamoxifen injection at E18.5. Arrows show representative cells stained with anti- p-Akt antibody. (**m**-**p**) BrdU (Bromodeoxyuridine) labeling of proliferating cells in the metaphysis of mice (tibia) control (**m**,**o**) and Kras^G12D^ (**n**,**p**) at P10 after Kras activation at E18.5. BrdU-positive cells are stained brown. (**q**) The BrdU index, calculated as the percentage of BrdU-labeled stromal cells, was significantly increased upon Kras^G12D^ activation. Stromal cells were defined as the non-hematopoietic cells that were not attached to the bone matrix. Data are represented as mean±S.E.M.; *n*=3, *P=0.002

**Figure 4:**
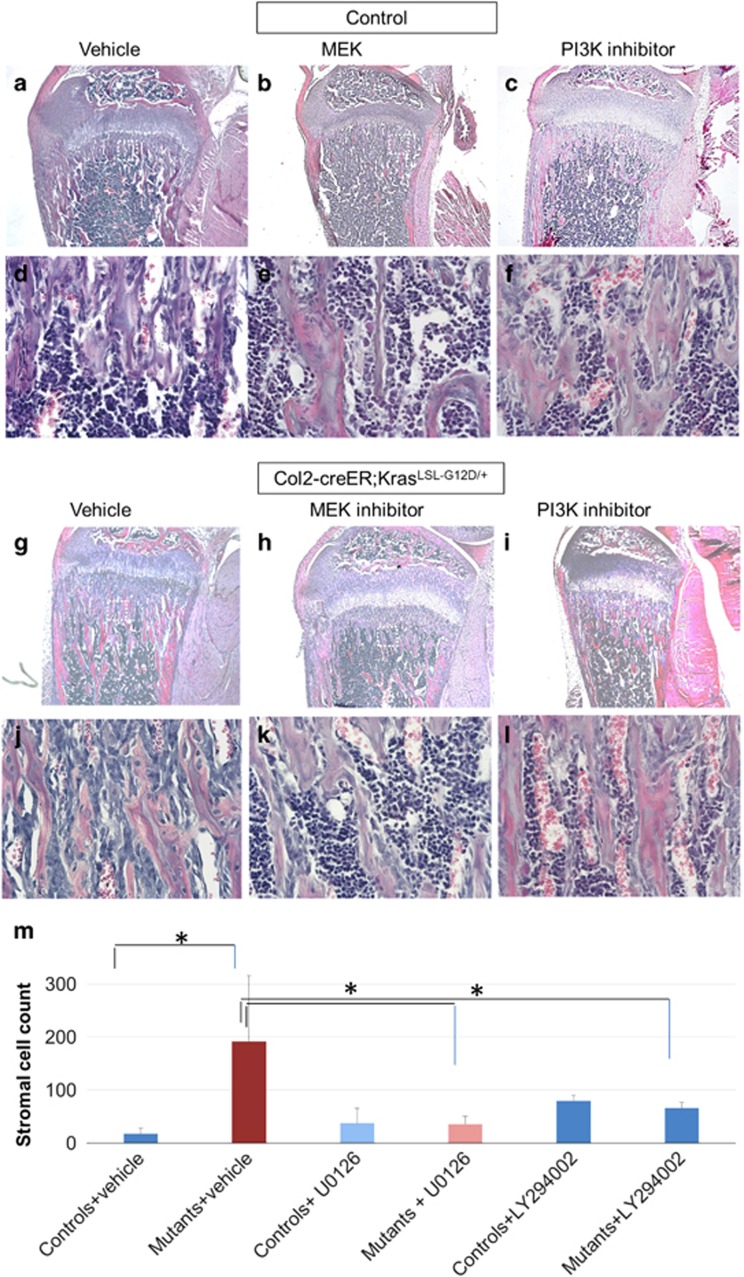
The MAPK and PI3K pathways are responsible for the increase in bone. (**a**-**f**) Hematoxylin/eosin-stained paraffin sections of tibias from Col2-creER or Kras^G12D^ (control) mice at P21 (tamoxifen injection at E18.5) treated with vehicle (methylcellulose) (**a**,**d**), MEK inhibitor (U0126; 5 mg/kg) (**b**,**e**), or PI3K inhibitor (LY294002 100 mg/kg) (**c**,**f**). (**g**-**l**) Hematoxylin/eosin-stained paraffin sections of tibias from Col2-creER;Kras^LSL-G12D/+^ mice at P21 after tamoxifen injection at E18.5 (**g**,**j**), with MEK inhibitor U0126 injections (**h**,**k**) or PI3K inhibitor LY294002 injections (**i**,**l**). After MEK or PI3K inhibition, mice harboring Kras^G12D^ show reduced trabecular bone and significantly smaller number of stromal cells than the vehicle-injected mutants. **a**,**b**,**c**, **g**, **h**, **i**: × 4 magnification. **d**, **e**, **f**,**j**, **k**, **l**: × 40 magnification. (**m**) Stromal cell counts in control and mutant mice injected with vehicle or MEK inhibitor or PI3K inhibitor. Data are represented as mean±S.E.M.; *n*=3, *P<0.05. Spindle-shaped cells, not attached to the bone matrix were counted in the center of secondary spongiosa in × 20 magnification

In addition to the increased bone mass, H/E staining revealed a substantial increase in the number of stromal cells between the trabeculae (Figures 3a and b, Supplementary Figures S2E, S2F, and Figure 4m). These stromal cells weakly expressed *Col1a1* while they were negative for the mature osteoblast marker, *Bglap* that encodes osteocalcin (Figures 3c and f).

As expected, Kras^G12D^ expression increased the number of cells that are positive for phosphorylated ERK1/2 (p-ERK) over time (Figures 3g and j). The PI3K pathway, another important pathway downstream of Kras, was also found activated in the stromal cells of Kras^G12D^ mice, as demonstrated by the increase in phosphorylation of Akt (p-Akt) (Figures 3k and l).

To assess the proliferation of stromal cells, BrdU assay was performed. As expected, the BrdU labeling index of the stromal cell population in the Kras^G12D^ mouse was increased at postnatal day P10 (Figures 3m and q).

Overactivation of Ras signaling influences cell survival. In order to test whether oncogenic Ras expression affects cell survival, a cell death assay (TUNEL assay) was performed to compare the apoptosis rate in the primary spongiosa of Kras^G12D^ mice and wild-type mice (Supplementary Figures S3E-S3G). Although a tendency for increased apoptosis in Kras^G12D^ mice was observed, the difference in cell death signals counted was not statistically significant (control *versus* mutant: 11±4.6 *versus* 19.7±4 *P*=0.07, *n*=3 each). This suggests that the increase in proliferation of stromal cells is the dominant mechanism for bone increase in the Kras^G12D^ mice.

We then wanted to address the question whether the effect of Kras overexpression in Col2-positive cells was age-specific. Activating Kras at P20 in Col2-positive cells caused a similar increase in bone and stromal cells, suggesting that Col2-positive cells are continuously present and serve as osteoprogenitor cells in postnatal bone (Supplementary Figures S3A-S3D).

### MAPK and PI3K are the main signaling pathways responsible for the increase in bone with Kras activation

Kras signals mainly through the mitogen-activated protein kinase (MAPK) and PI3K pathways.^11^ In order to investigate whether activation of these signaling pathways was responsible for the bone phenotype of Kras mice, we pharmacologically inhibited the ERK and PI3K pathways. We injected U0126, an inhibitor of ERK kinase (MEK), or LY294002, a PI3K inhibitor from E18.5 through P21. Both MEK inhibition (Figures 4h and k) and PI3K inhibition (Figures 4i and l) partially, but significantly, rescued the phenotype in Col2-creER; Kras^G12D^ mice injected with tamoxifen at E18.5, as demonstrated by the decreased stromal cell number and trabecular bone (Figure 4h, i, k, l, m and comparison with figures 4g, j). In both MEK inhibition and PI3K inhibition, their downstream molecules (p-ERK and p-Akt signals respectively) were suppressed after the treatment with inhibitors (Supplementary Figures S4A-S4F). These findings suggest that activation of both MAPK and PI3K pathways contribute to the bone phenotype of Kras^G12D^ mice.

### Kras^G12D^ expression in Col1-positive cells does not increase stromal cells or bone mass

Next, we investigated the effect of Kras activation in lineage-committed osteoblasts. We used Osx-creER and Col1-creER to target osteoblasts in early and late differentiation stages, respectively. A single dose of tamoxifen was injected into mice at E18.5 to induce expression of Kras^G12D^ and Tomato in Osx-positive or Col1-positive cells.

**Figure 5:**
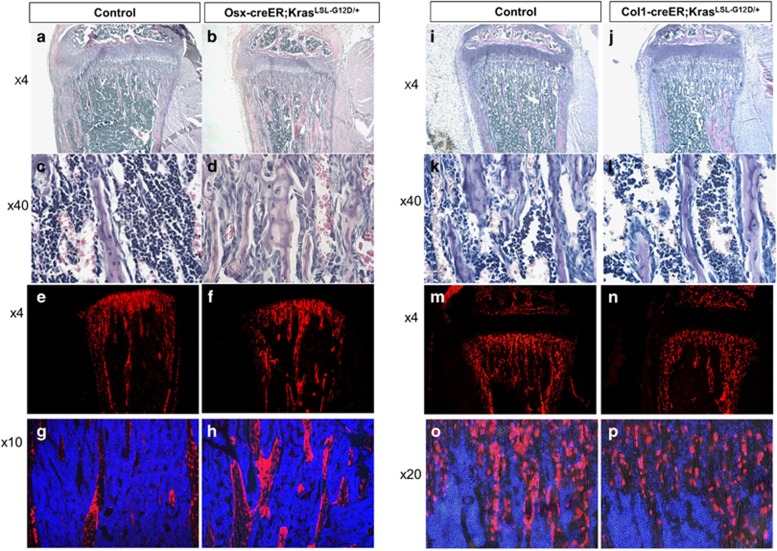
Overexpression of Kras in Osx cells perinatally causes a milder increase in bone and stromal cells, while in Col1 cells it has no effect (see also Supplementary figure S4). (**a**-**b**) Hematoxylin/eosin-stained paraffin sections of tibias from P28-old Osx-creER mice control (**a**,**c**) and Kras^G12D^ (**b**,**d**) after Kras activation at E18.5. **a**,**b**: × 4 magnification, **c**,**d**: × 40 magnification. (**e**-**h**) Cryosections showing tomato-positive cells in P28-old Osx-creER mice control (**e**,**g**) and Kras^G12D^ (**f**,**h**) after tamoxifen injection at E18.5. **e**,**f** × 4 magnification; **g**,**h** × 10 magnification. Blue, DAPI; red, Tomato red fluorescent protein. (**i**-**l**) Hematoxylin/eosin-stained paraffin sections of tibias from P28 Col1-creER mice control (**i**,**k**) and Kras^G12D^ (**j**,**l**) after tamoxifen injection at E18.5. **i**,**j**: × 4 magnification, **k**,**l**: × 40 magnification. (**m**-**p**) Cryosections showing tomato-positive cells in P28 Col1-creER mice with wild-type Kras (**m**,**o**) and Kras^G12D^ (**n**,**p**) after tamoxifen injection at E18.5. **m**,**n**: × 4 magnification, **o**,**p**: × 20 magnification. Blue, DAPI; red, Tomato red fluorescent protein. For both the Osx model and Col1 model *n*=5 mice per group

Kras^G12D^ expression in Osx-positive cells caused a phenotype similar to, but somewhat milder than, that of the Col2-creER model (Figures 5a and d). Tomato-positive Osx-positive cells and their descendants were found in both bone and stromal compartments (Figures 5e and h).

In contrast, activating Kras in the Col1-positive cells caused no significant changes in bone or stromal cells at P28 (Figures 5i and l), despite that Kras^G12D^ expression significantly increased ERK phosphorylation in osteoblasts (Supplementary Figures S5A-S5D). Consistent with this result, there was no significant increase in tomato-positive Col1-positive cell descendants (Figures 5m and p). These results suggest that Ras activation has no stimulatory effect in differentiated osteoblasts.

## Discussion

In this study, we investigated the role of Ras signaling in three different cell populations of the osteoblast lineage, that is, Col2-, Osx- and Col1-positive cells, during bone development.

Kras overactivation in Col2-positive or Osx-positive cells in bone at E18.5 led to a significant increase in number of their descendant cells and trabecular bone mass. In contrast, Kras overactivation in Col1-positive cells did not show significant effects in cell number or bone mass, suggesting that Col2- and Osx-positive cells, but not Col1-positive cells, include proliferative osteoblast progenitors. This study also demonstrates that Ras signaling is an important positive regulator of proliferation of early osteoprogenitor cells.

Multiple signaling molecules, including receptor tyrosine kinases (RTKs),^12^ have been implicated in regulation of proliferation and survival of bone progenitor cells.^13, 14^ Kras, a small GTPase activated by RTKs, triggers downstream effector pathways, such as the MAPK (mitogen-activating protein kinase), PI3K (Phosphatidylinositol 3'-kinase) and Ral-GEF (Ras-like small GTPases) pathways, to regulate cellular functions including proliferation, differentiation and apoptosis.^15^ Multiple Ras-activating molecules, including fibroblast growth factors (FGFs)/FGF receptors,^16^ epidermal growth factors (EGFs)/ EGF receptors^17^ and insulin-like growth factors (IGFs) and their receptors,^18^ have been implicated in regulating bone development.

Ras signaling has been shown to increase stem/progenitor cell proliferation in multiple tissues. Kras activation has been shown to increase proliferation of bone marrow hematopoietic cells.^19, 20, 21^ Additionally an oncogenic Kras increases proliferation of mouse embryonic fibroblasts^22^ and facilitates self-renewal of a subtype of alveolar stem cells.^23^ Additionally Ras activation by partial loss of Nf1 gene in osteoprogenitor cells was found to increase proliferation and cause premature apoptosis.^24^ In the present model apoptosis was not found significantly increased; thus, we conclude that the increase in proliferation is the dominant effect of Kras activation in osteoprogenitor cells.

However, there have been relatively limited studies that investigate the role of Ras-MAPK signaling pathway in osteoblast progenitor cells in bone *in vivo*. It has been shown that the ERK signaling pathway, a major downstream mediator of Ras, in mesenchymal progenitor cells promotes osteoblast differentiation and bone formation during early skeletal development.^25^ Nonetheless, the proliferative effect of ERK signaling specifically in osteoprogenitor cells has not been addressed. In another study in which a dominant negative or a constitutively active MEK1 was expressed in relatively mature osteoblasts, ERK signaling stimulated osteoblast differentiation without obvious changes in proliferation.^26^ The result of this study is consistent with our finding that Kras overactivation in Col1-positive mature osteoblasts had little effect in bone mass or stromal cell proliferation. In our study, we found that ERK inhibition significantly reduced the number of stromal cells in Kras^G12D^ mice, suggesting that ERK overactivation is, at least in part, responsible for the proliferative effect of Kras^G12D^ expression in Col2-positive osteoprogenitors. We also found that PI3K inhibition partially reduced stromal cells and partially rescued the bone phenotype caused by Kras^G12D^ expression. Thus our study demonstrates that in addition to the ERK pathway, the PI3K pathway also plays an important role in osteoprogenitor proliferation.

To investigate the pathways downstream of Kras we injected mice with small molecule inhibitors of MEK and PI3K. With the dosage and timeframe of these experiments we did not observe significant changes in the mouse body size or condition. The wild-type control mice injected with MEK inhibitor showed no appreciable changes in bone.

Over-expression of active Kras in Col2-Cre-positive cells is not physiologically present during development, and therefore our findings do not necessarily demonstrate that endogenous Ras signaling is a physiologic regulator of osteoprogenitor cells. Nevertheless, using this system, we are able to show that Col2-positive or Osx-positive cells, but not Col1-positive cells include osteoprogenitor cells that can be discriminated by responses to Ras signaling. Additionally these experiments show that MAPK and PI3K pathways activation in the above cell populations can activate bone formation.

The loss of balance between bone formation and bone resorption results in osteoporosis.^27^ Since treatment options to increase bone formation are currently limited,^28^ novel bone anabolic therapies are highly desired. Our study shows that stimulating Ras signaling in endogenous osteoprogenitor cells can be a valid treatment strategy for osteoporosis.

## Materials and Methods

### Mice

Col2a1-creERT2,^8^ Osx-creERT2,^3^ Col1-creERT2,^9^ and Cre-dependent Kras transgenic, loxP-stop-loxP-Kras^G12D^^10^ mice have been described elsewhere. The Cre reporter, Rosa26-loxP-stop-loxP-tdTomato mice were acquired from Jackson Laboratory (Bar Harbor, ME, USA).^7^ All procedures were conducted in compliance with the Guideline for the Care and Use of Laboratory Animals approved by Massachusetts General Hospital's Institutional Animal Care and Use Committee (IACUC). Mice were in a mixed genetic background. Comparisons were made between littermates. Tamoxifen injections were given i.p. at a dose of 0.1 mg/g to pregnant females as described elsewhere.^4^

### Histology

For histological analysis mice were dissected, fixed in 10% formalin overnight and decalcified in 10% EDTA for a period ranging from 1 to 14 days, depending on the age of the mice. For H/E staining, BrdU staining, immunohistochemistry and *in situ* hybridization, samples were paraffin processed and cut. For fluorescent reporter analysis decalcified samples were cryoprotected in 30% sucrose/ phosphate buffer saline (PBS) solutions then in 30% sucrose/PBS:OCT (1:1) solutions, each overnight at 4 °C. Samples were embedded in OCT compound (TissueTek, Sakura, Torrance, CA, USA) and transferred to dry ice to solidify OCT. Samples were cryosectioned at the thickness of 15 *μ*m using a cryostat (Leica CM1850). Nikon Eclipse E800 microscope was used for imaging. Images were merged with Spot Advanced Software.

### Immunohistochemistry and *in situ* hybridization

Immunohistochemistry analysis for phosphorylated ERK (phospho-ERK) was performed as previously described using the Perkin Elmer TSA biotin system kit.^29^ The primary antibody directed against phospho-p44/42 (ERK1/2) (Cell Signaling, Danvers, MA, USA, catalog number:#43700) was diluted 1:300 and the secondary biotinylated anti-rabbit antibody (Vector, Burlingame, CA, USA, catalog number #BA1000) was diluted 1:300. Anti p-Akt (Cell Signaling, catalog number #2965) antibody was diluted 1:500. ISH for type 1 collagen and osteocalcin was performed according to published protocols.^30^ For all immunostaining experiments, samples from three mice per group were analyzed.

### Cell proliferation assay

For BrdU labeling 50 *μ*g of BrdU per gram of body weight was intraperitoneally injected into mice 2 h before euthanasia. BrdU was detected using the BrdU *in situ* staining kit (invitrogen, Waltham, MA, USA). The BrdU labeling index was calculated as the ratio of BrdU-positive nuclei over total nuclei of stromal cells of the metaphyseal tibia.

### TRAP staining

Slides were deparaffinized and rehydrated. TRAP reagent consisted of 6 ml 50 mM tatrate in acetate buffer (PH 5.0), 0.5 mg Naphthol As-Mx, 50 *μ*l N,N dimethylamide and 3 mg fast red violet LB. TRAP reagent was added for 5–10 min and once satisfactory color was observed, slides were washed with water and then with PBS. At the end the slides were mounted with water-based medium.

### Cell death assay (TUNEL assay)

Cell death was evaluated on sections using an *in situ* cell death detection kit (Roche, Branford, CT, USA) according to the manufacturer's instructions.

### MicroCT analysis

A high-resolution desktop micro-tomographic imaging system (*μ*CT40, Scanco Medical AG, Basserdorf, Switzerland) was used to assess trabecular and cortical in the proximal metaphysis and diaphysis of the tibia, respectively. Scans were acquired and analyzed as previously described.^31^ Image acquisition and analysis were performed according to the JBMR guidelines for the use of *μ*CT in rodents.^32^

To assess the trabecular architecture, transverse CT slices were evaluated in a 1500 *μ*m (150 slices) region beginning 750 *μ*m below the proximal growth plate and extending distally. The trabecular bone region was identified by semi-manually tracing with the assistance of an auto-thresholding software algorithm. A mineral density threshold of 225 mgHA/cm^3^ was used for all images to segment bone from soft tissue.

### Rescue experiments- inhibitors

The MEK1 inhibitor (U0126) and the PI3K inhibitor (LY294002) were purchased from Selleckchem (Houston, TX, USA). Inhibitors were first dissolved in DMSO according to the manufacturer's instructions, then diluted into PBS, and injected daily into pregnant as well as nursing mothers intraperitoneally from E18.5 through P9.5. Subsequently, the inhibitors were injected to pups. MEK inhibitor was injected at a dose of 5 mg/kg per day. PI3K inhibitor was injected at a dose of 100 mg/kg four times a week.

## Abbreviations

ERK: extracellular signal-regulated kinase
p-ERK: phosphorylated extracellular signal-regulated kinase
MAPK: mitogen-activated protein kinases
MEK: MAPK/ERK kinase
PI3K: phosphoinositide 3 kinase
GTP: guanosine triphosphate
TRAP: tartrate-resistant acid phosphatase
*μ*CT: microcomputed tomography
H/E: hematoxylin eosin staining
TUNEL: terminal deoxynucleotidyl transferase (TdT) dUTP nick-end labeling
RTK: receptor tyrosine kinase
Ral GEF: Ras-like small GTPases
FGF: fibroblast growth factor
EGF: epidermal growth factor
IGF: insulin growth factor
IACUC: institutional animal care and use committee
EDTA: ethylenediaminetetraacetic acid
BrdU: Bromodeoxyuridine
DAPI: 4',6-diamidino-2-phenylindole
ISH: *in situ* hybridization
S.E.M.: standard error of the mean
