# Supplementary figures and images for: Ras signaling regulates osteoprogenitor cell proliferation and bone formation
Source: Cell Death Dis. 2016 Oct 13;7(10):e2405–. doi: 10.1038/cddis.2016.314 (PMC5133981; doi:10.1038/cddis.2016.314)

Col2CreERt(Tg/-) R26Tom(+/-)

P1.5

P10

P20

X4

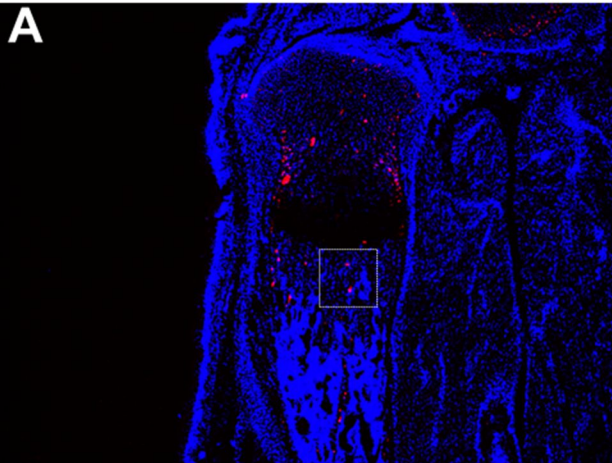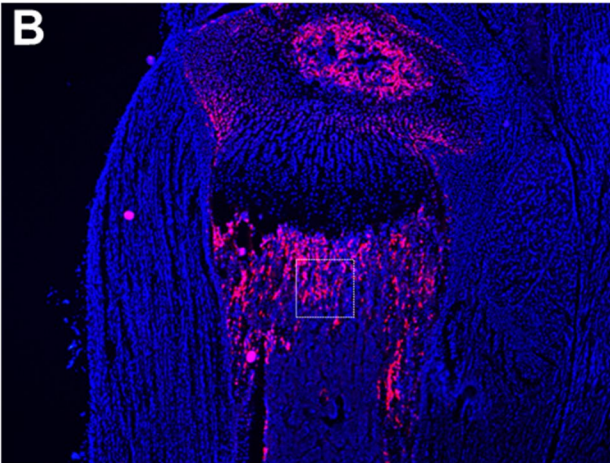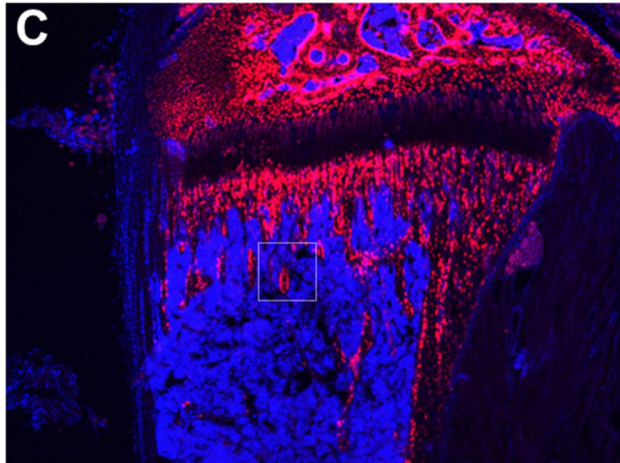

X40

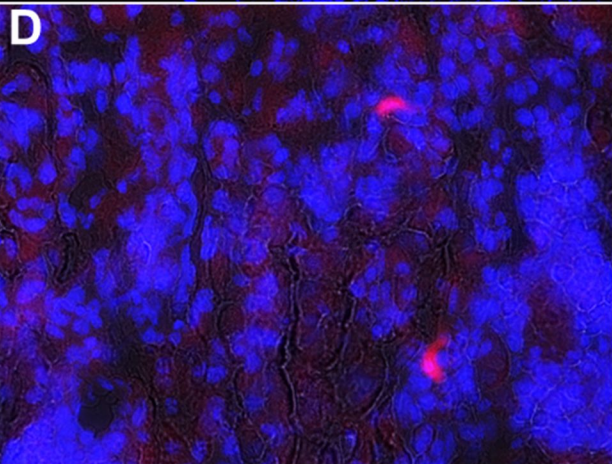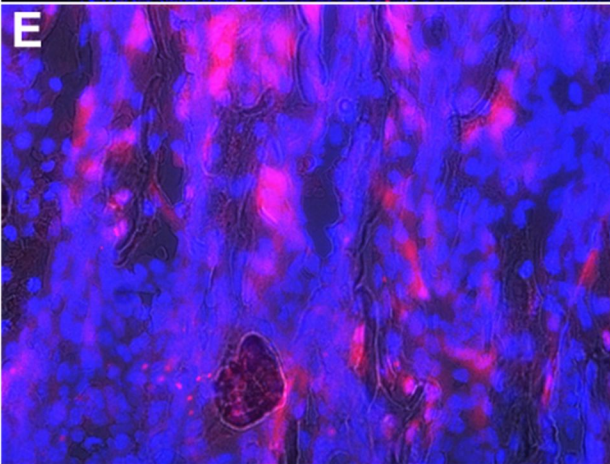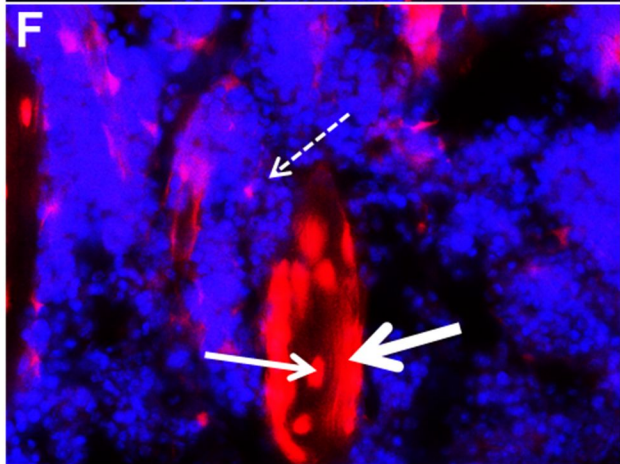

OsxCreERt(Tg/-) R26Tom(+/-)

P4.5

P28

x4

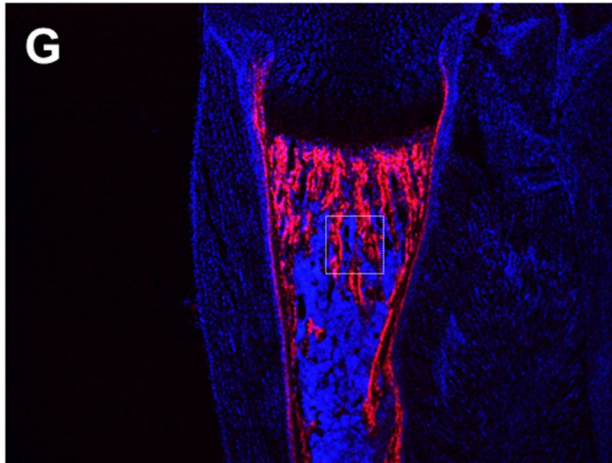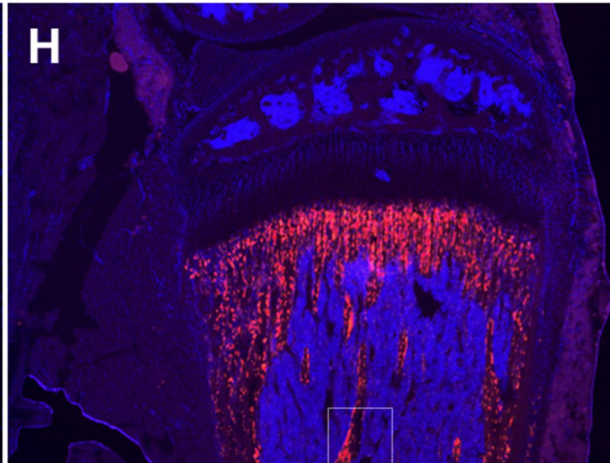

X40

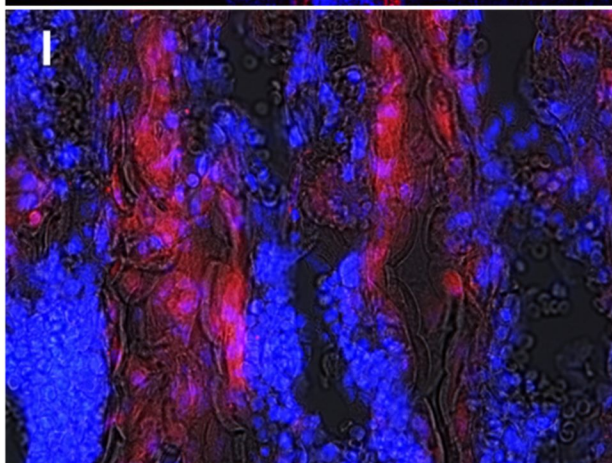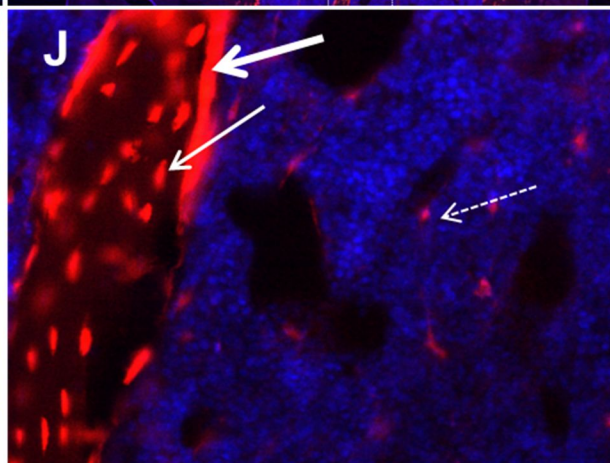

Col1CreERt(Tg/-) R26Tom(+/-)

P1.5

P28

X4

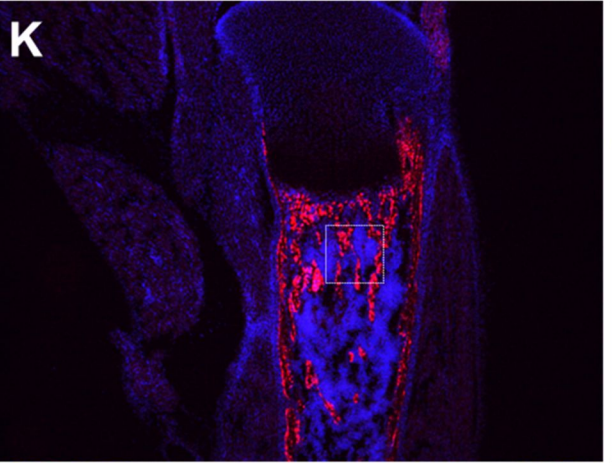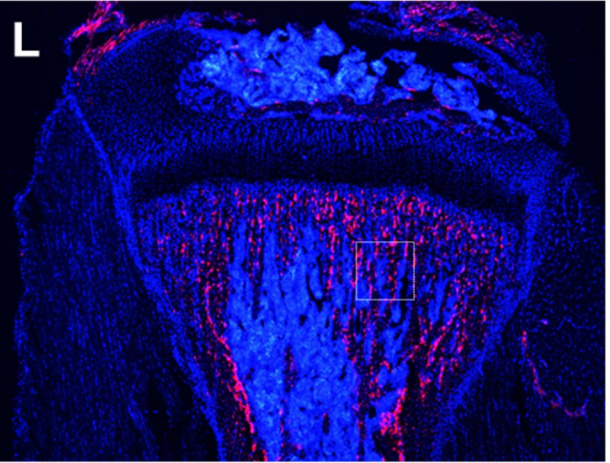

X40

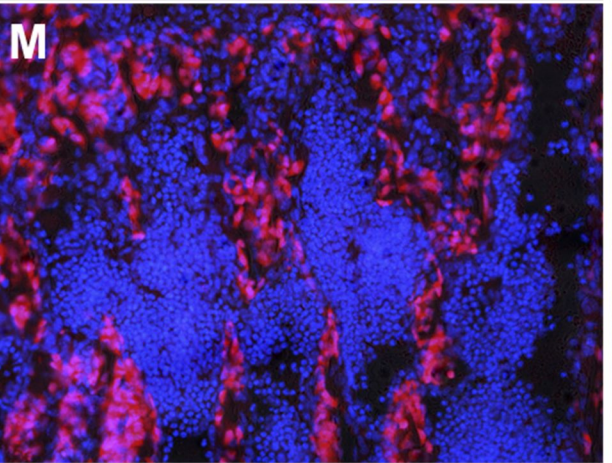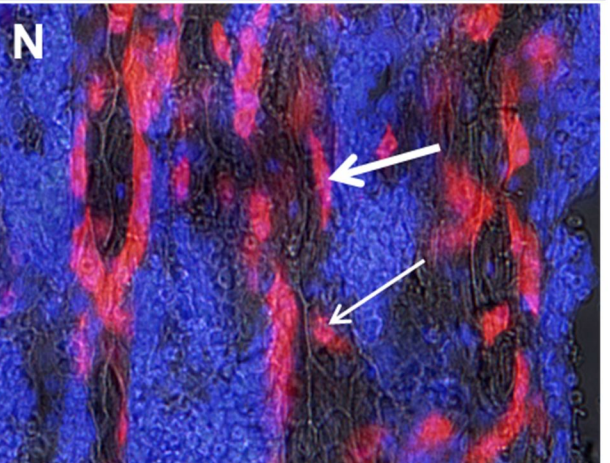

Supplement: Supplementary Figure S1 [file cddis2016314x1.pdf]

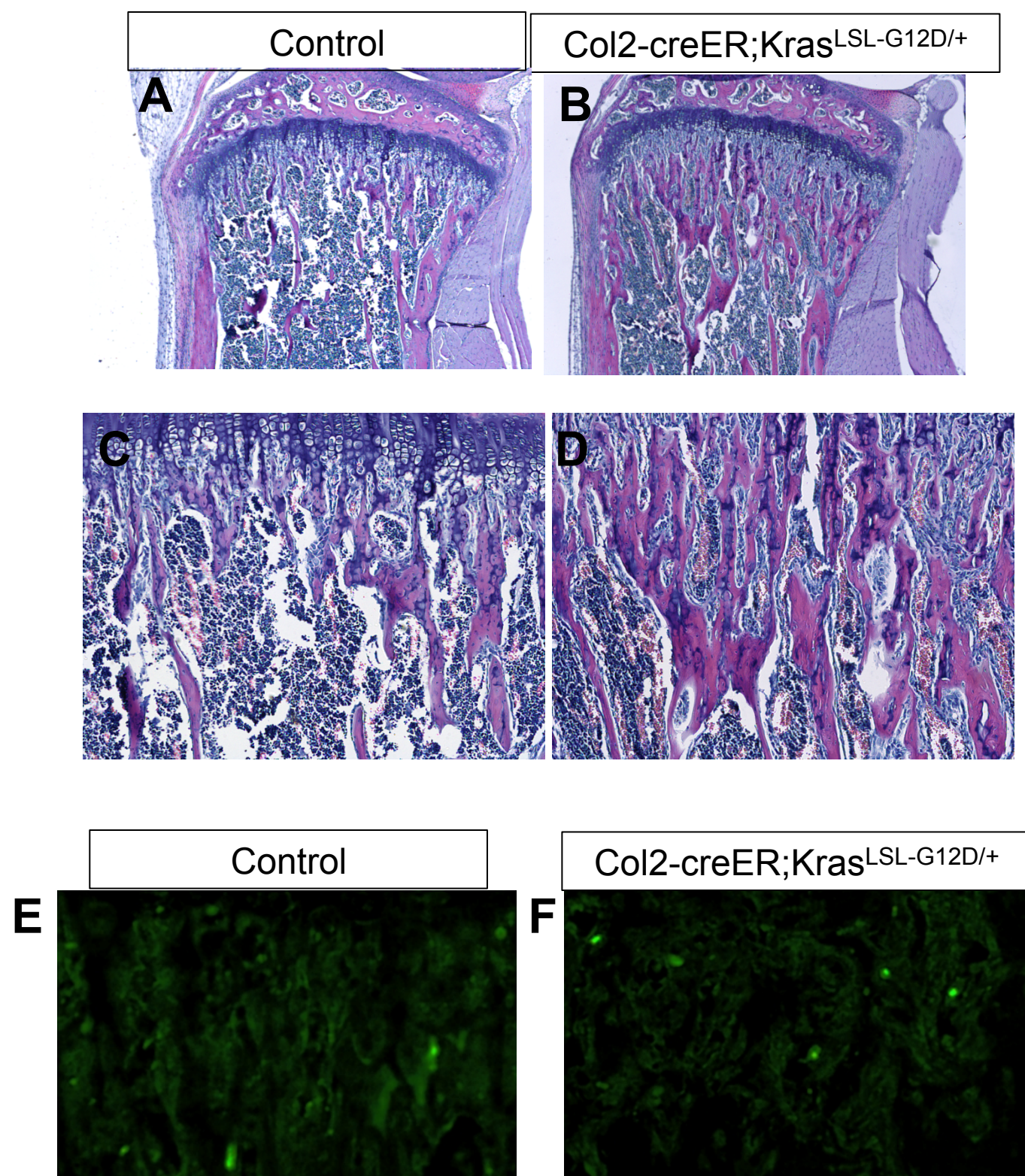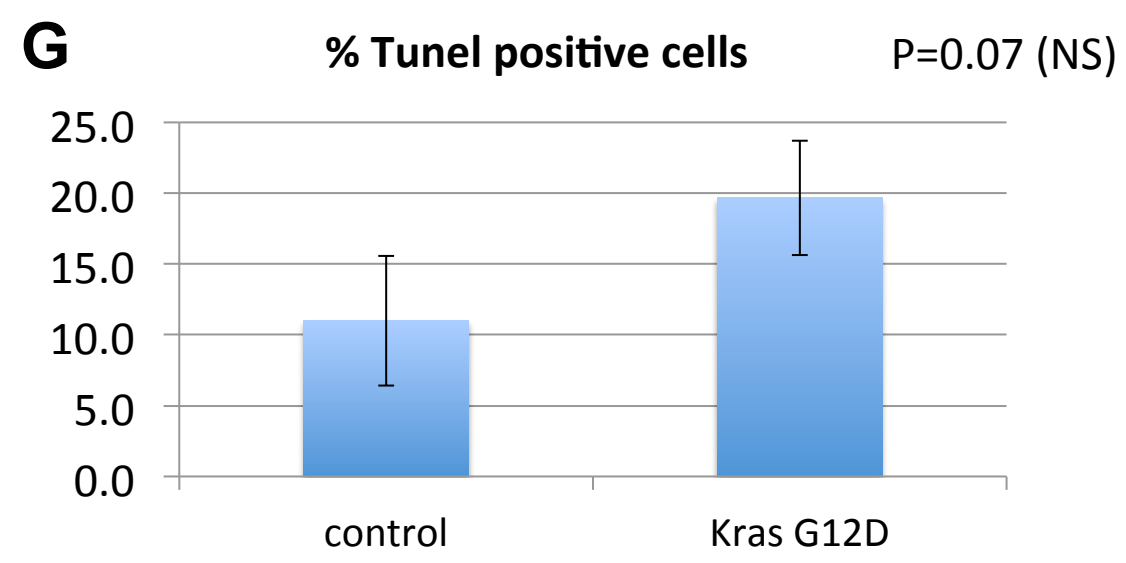

Supplement: Supplementary Figure S3 [file cddis2016314x3.pdf]

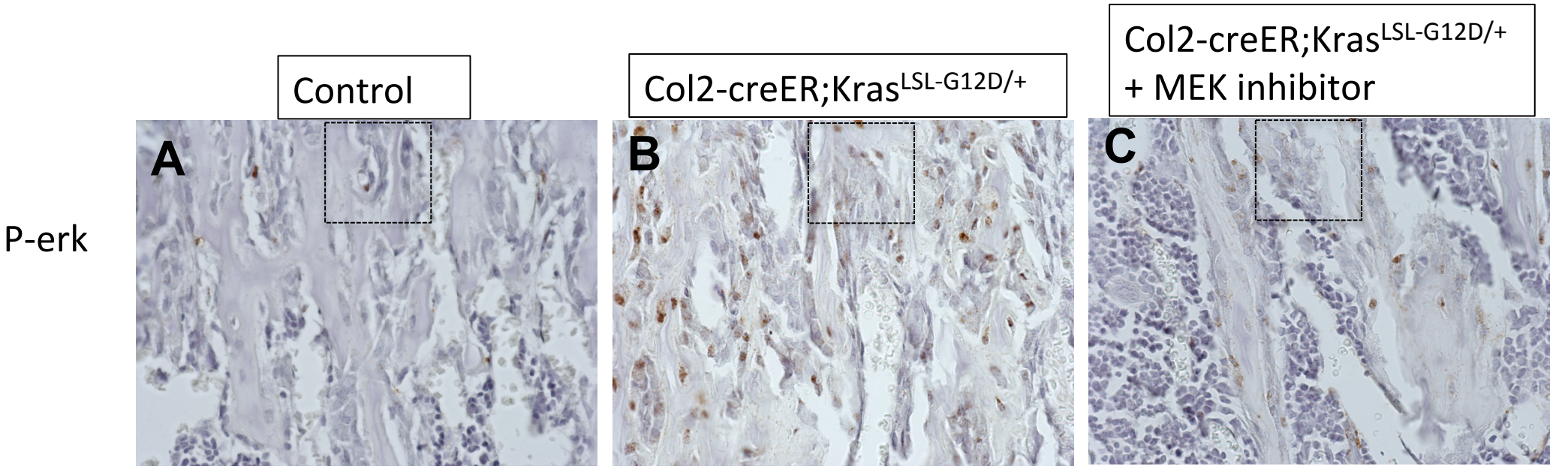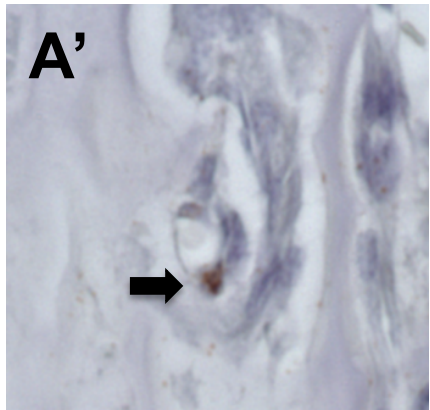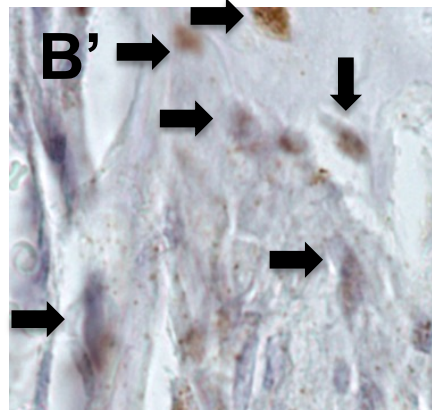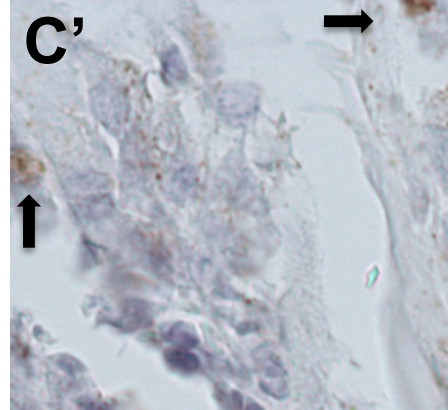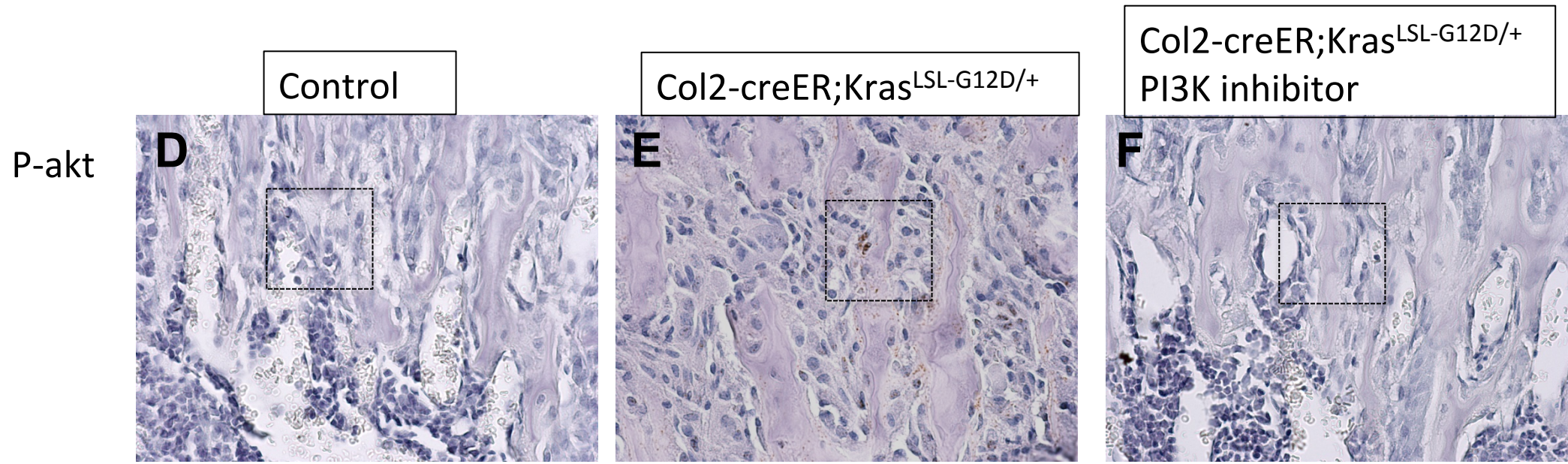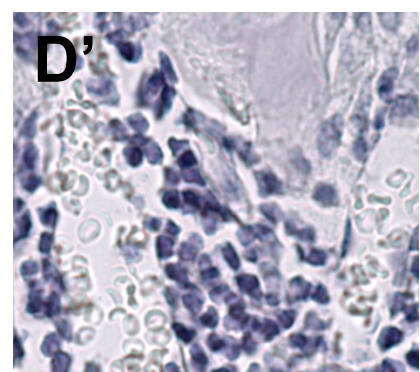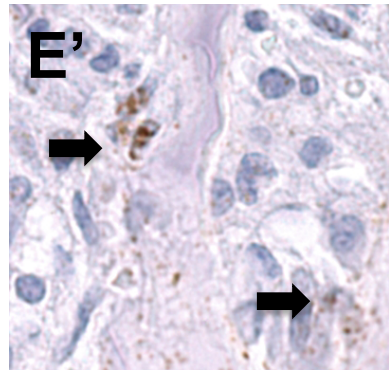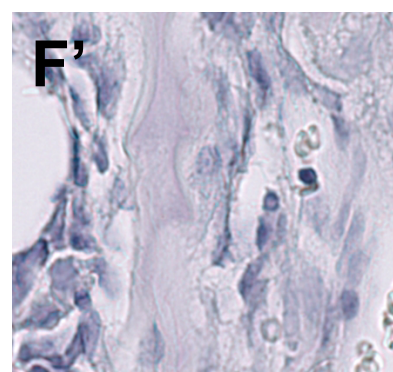

Supplement: Supplementary Figure S4 [file cddis2016314x4.pdf]

**WT**

**Col1-creER;Kras<sup>LSL-G12D/+</sup>**

**A**

**B**

x4

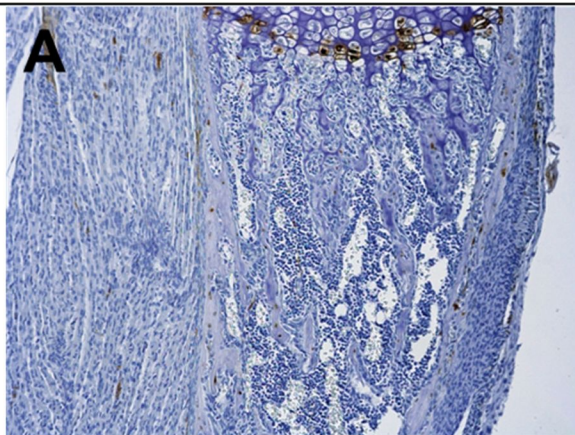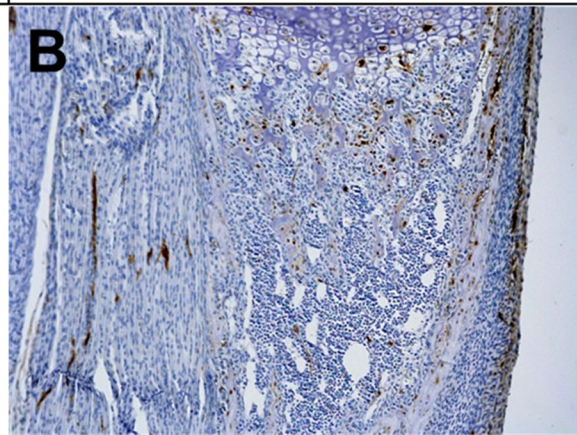

**C**

**D**

x40

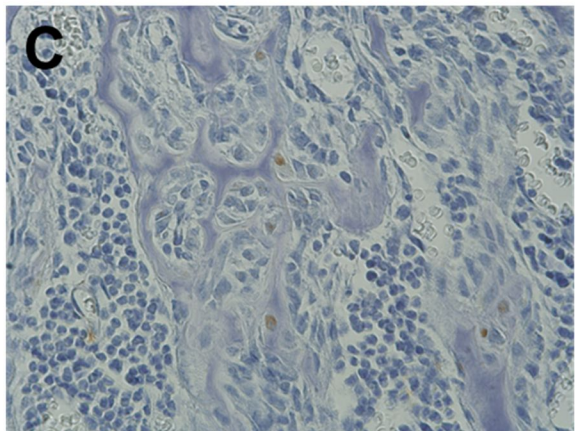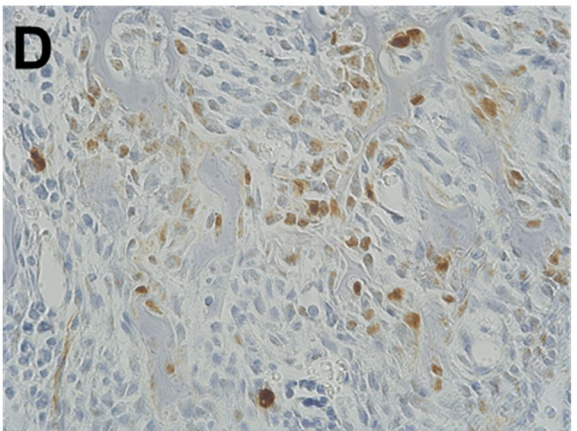

Supplement: Supplementary Figure S5 [file cddis2016314x5.pdf]
