# Supplementary material for: Ras signaling regulates osteoprogenitor cell proliferation and bone formation
Source: Cell Death Dis. 2016 Oct 13;7(10):e2405–. doi: 10.1038/cddis.2016.314 (PMC5133981; doi:10.1038/cddis.2016.314)
Supplement: Supplementary Figure S2 [file cddis2016314x2.pdf]

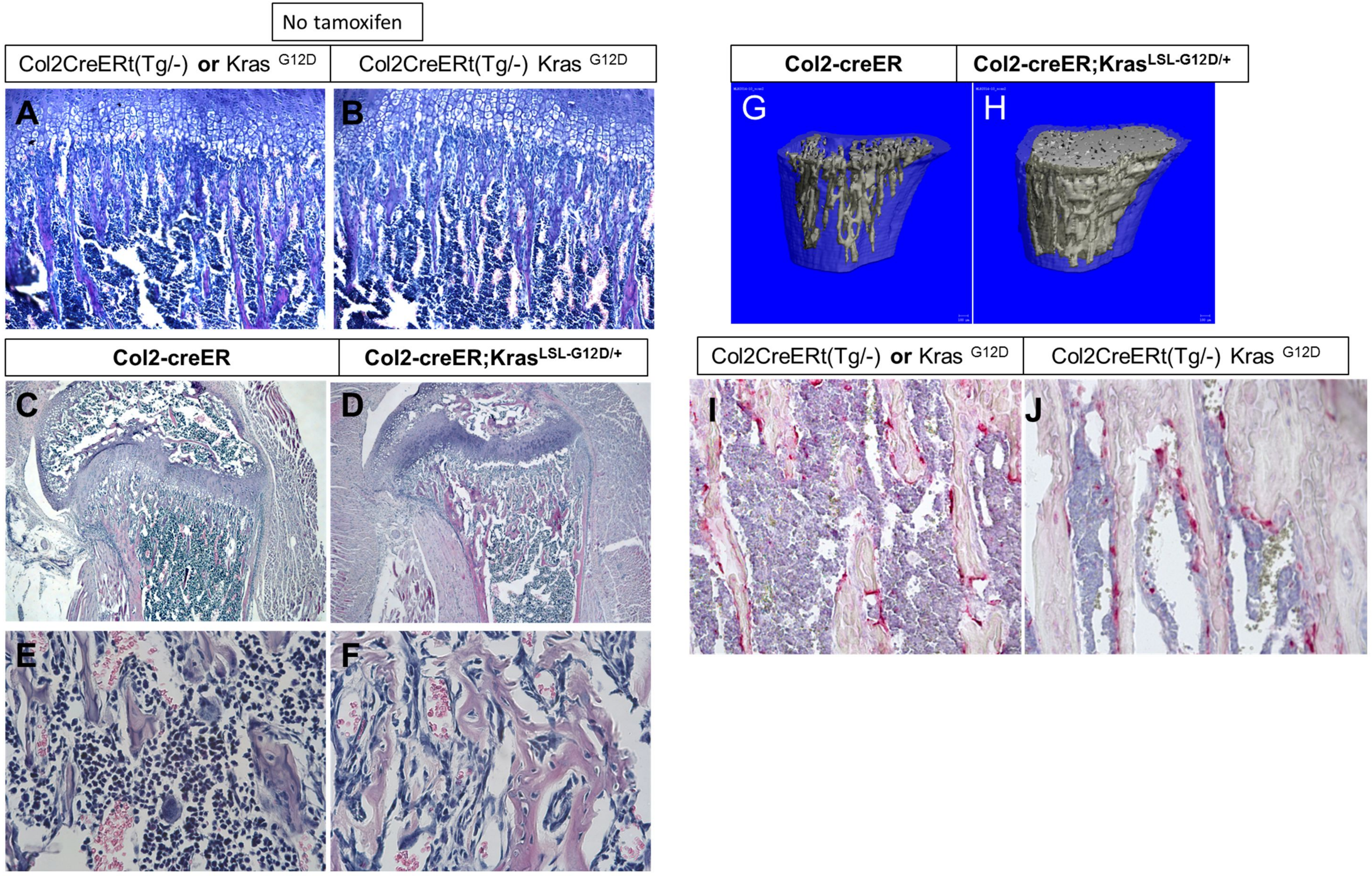
No tamoxifen
Col2CreERt(Tg/-) or Kras G12D
Col2CreERt(Tg/-) Kras G12D
A
B
Col2-creER
Col2-creER;Kras LSL-G12D/+
G
H
Col2-creER
Col2-creER;Kras LSL-G12D/+
C
D
E
F
Col2CreERt(Tg/-) or Kras G12D
Col2CreERt(Tg/-) Kras G12D
I
J
