# Supplementary material for: Ras signaling regulates osteoprogenitor cell proliferation and bone formation
Source: Cell Death Dis. 2016 Oct 13;7(10):e2405–. doi: 10.1038/cddis.2016.314 (PMC5133981; doi:10.1038/cddis.2016.314)
Supplement: Supplementary Figure Legends [file cddis2016314x6.doc]

**Supplemental figures**

Figure S1 (associated with Figure 1):

**A-F**. Cryosections of the tibia of Col2-creER;R26^tomato^ mice pulsed with tamoxifen at E18.5. Col2-positive cells were genetically labeled with Tomato (red fluorescent protein) at age E18.5 and their cell fate was chased to the ages of P1.5, P10 and P28. **A-C**: x4 magnification, **D-F**: x40 magnification. White arrow: osteocyte, thick white arrow: osteoblast, white dashed arrow: stromal cell. D-F are magnified pictures of boxes in A-C respectively.

**G-J.** Cryosections of the tibia of Osx-creER;R26^tomato^ mice pulsed with tamoxifen at E18.5. Osx-positive cells were labeled with Tomato at E18.5 and their cell fate was chased to the ages of P4.5 and P28. **G-H** : x4 magnification, **I-J**: x40 magnification of boxes in G,H respectively. White arrow: osteocyte, thick white arrow: osteoblast, white dashed arrow: stromal cell.

**K-N**. Cryosections of the tibia of Col1-creER;R26^tomato^ mice pulsed with tamoxifen at E18.5. Col1 positive cells were labeled with Tomato at E18.5 and their cell fate was chased to the ages of P1.5 and P28. **K-L**: x4 magnification, **M-N**: x40 magnification of boxes in K,L respectively. White arrow: osteocyte, thick white arrow: osteoblast.

Red: Tomato, blue: DAPI.

Figure S2 (associated with Figure 2): **Expression of Kras^G12D^ in Col2-positive cells increases number of their descendants and trabecular bone mass**

**A-B** Hematoxylin/eosin-stained paraffin sections of tibias from P28-old Col2-creER mice control (A) or Kras^G12D^ (B) without tamoxifen injection (Kras was not activated) (x4 magnification).

**C-F**. Hematoxylin/eosin-stained paraffin sections of the humerus from P28-old Col2-creER mice control (C,E) or Kras^G12D^ (D,F) after tamoxifen injection at E18.5. C,D x4 magnification; E,F x40 magnification.

**G-H** μCT 3D pictures of the tibial mataphysis from P28-old Col2-creER mice control (G) or Kras^G12D^ (H) after tamoxifen injection at E18.5.

**I-J** Tartrate-resistant acid phosphatase (TRAP)-stained paraffin sections of the secondary spongiosa from P28-old Col2-creER mice control (I) or Kras^G12D^ (J) after tamoxifen injection at E18.5. n=3

Figure S3 (associated with Figure 3): **Activation of a Kras oncogene in Col2 cells at a post-natal age increases stromal cell numbers**

**A-D.** Hematoxylin/eosin-stained paraffin sections of tibias from P50-old Col2-creER mice with control (A,C) and Kras^G12D^ (B,D) after tamoxifen injection at P20.

**E-G**. The TUNEL assay on paraffin sections of control and Kras^G12D^ ,mice. The primary spongiosa region, corresponding to figures S3C-D is shown (x10 magnification). control vs mutant: 11±4.6 vs 19.7±4 p=0.07, n=3 each.

Figure S4 (associated with Figure 4): **The MAPK and PI3K pathways are responsible for the increase in bone.**

**A-C.** Immunostaining for p-ERK on tibia sections from control (A) or Col2-creER;Kras^LSL-G12D/+^ (B) or Col2-creER;Kras^LSL-G12D/+^ (C) mice at P21 treated with MEK inhibitor (U0126; 5mg/kg). Tamoxifen was injected at E18.5. x40 magnification. **A’-C’** are magnifications of boxes in figures A-C.

**D-F.** Immunostaining for p-Akt on tibia sections from control (D) or Col2-creER;Kras^LSL-G12D/+^ (E) or Col2-creER;Kras^LSL-G12D/+^ (F) mice at P21 treated with PI3K inhibitor (LY294002 100 mg/kg). Tamoxifen was injected at E18.5. x40 magnification. **D’-F’** are magnifications of boxes of in figures D-F.

Figure S5 (associated with Figure 5): **Overexpression of Kras**^G12D^ **in Col1-positive cells increases p-ERK but it has no effect in bone and stromal cells.**

**A-D.** Immunohistochemistry for p-ERK at P1.5 in the secondary spongiosa in tibia of Col1-creER mice with control (A,C) or Kras^G12D^ (B,D) after tamoxifen injection at E18.5. A,B x4 magnification; C, D x40 magnification.
